# Reproductive failure in moose (*Alces alces*) due to embryonic mortality and unfertilized oocytes

**DOI:** 10.1007/s13364-013-0173-6

**Published:** 2013-12-15

**Authors:** Jonas Malmsten, Anne-Marie Dalin

**Affiliations:** 1 Division of Reproduction, Department of Clinical Sciences, Swedish University of Agricultural Sciences, Box 7054, SE-750 07 Uppsala, Sweden; 2 Department of Pathology and Wildlife Diseases, National Veterinary Institute, SE-751 89 Uppsala, Sweden

**Keywords:** Conception rate, Embryonic mortality, Moose, Ovulation, Pregnancy failure, Reproduction

## Abstract

Knowledge on reproductive success is vital for successful management of large ungulates and is often measured by means of observing surviving offspring. In harvested ungulates, postmortem investigations of reproductive organs are used to estimate reproductive potential by obtaining ovulation rates and fetus numbers. However, there are differences in numbers of offspring observed, fetal/embryo counts, and ovulation rates. We hypothesize that the discrepancy between estimated reproductive potential and reproductive outcome in large ungulates is not only due to ova loss but also due to embryonic mortality. We investigated reproductive status in early pregnancy by sampling hunter-harvested moose (*Alces alces*) in southern Sweden from 2007 to 2011. In all, 213 reproductive organs were examined postmortem, and in confirmed pregnant moose (*n* = 53), 25 % (19 of 76) embryos were nonviable and 6 % of ova was unfertilized. The discrepancy between the ovulation rate of all pregnant moose (1.49) and the number of expected offspring per pregnant female, when embryonic mortality and unfertilized oocytes were accounted for (1.08), was 27.5 %. An association between inflammation of the inner mucous membrane (endometritis) of the moose's uterus and embryonic mortality was observed. This is the first comprehensive report of embryonic mortality and endometritis in moose. The observed discrepancy between ovulation rates and early embryonic development/survival shows that ovulation rates are indicative but not accurate estimates of moose reproductive rate. The use of ovulation rates as a sole estimator of future offspring rates may lead to an overharvest of a managed moose population.

## Introduction

For intensively managed large ungulates, knowledge about reproductive performance (individual and population fecundity) is vital for successful management and population sustainability. In many wild, large ungulate species, knowledge of reproduction is to a large extent based on research in captive animals, kept under restricted conditions, which may not reflect the situation of a natural habitat. To obtain further knowledge about reproduction in wild animals, there is a need to study specimens in the wild. In this respect, obtaining large amounts of samples is feasible in species subjected to harvest. In moose (*Alces alces*), reproductive performance is usually measured by observing numbers of surviving offspring after the summer period (calf per adult female ratio, Ericsson and Wallin 1999). Reproductive potential as well as individual and population fecundity (population production of offspring) among female moose can also be obtained by performing postmortem examinations of reproductive organs. In general, ovulation rates and/or embryo/fetal counts are used as the standard measurement. Some reports indicate that embryonic mortality and unfertilized oocytes (ova loss) account for a discrepancy between the number of fetuses and ovulation rates in harvested females (Markgren 1969; Schwartz and Hundertmark 1993). Though rarely reported, disorders of the reproductive system in wild, large ungulates could affect reproductive success and thus population viability. Such disorders may include embryonic mortality (of various origin), diseases of the reproductive tract (infectious and noninfectious), and loss of ova/oocytes. The aim of this study was to determine the discrepancy between ovulation rates and early pregnancy loss (due to embryonic mortality and unfertilized oocytes) in free-ranging Scandinavian moose by postmortem examination of reproductive organs from hunter-harvested females. We hypothesize that loss of ova is not only attributed to non-fertilization of oocytes (ova loss) but also dependent on embryonic mortality and survival. Also, we hypothesize that there is a discrepancy between ovulation rates and embryo survival rates.

## Materials and methods

### Sampling of moose

**Fig. 1:**
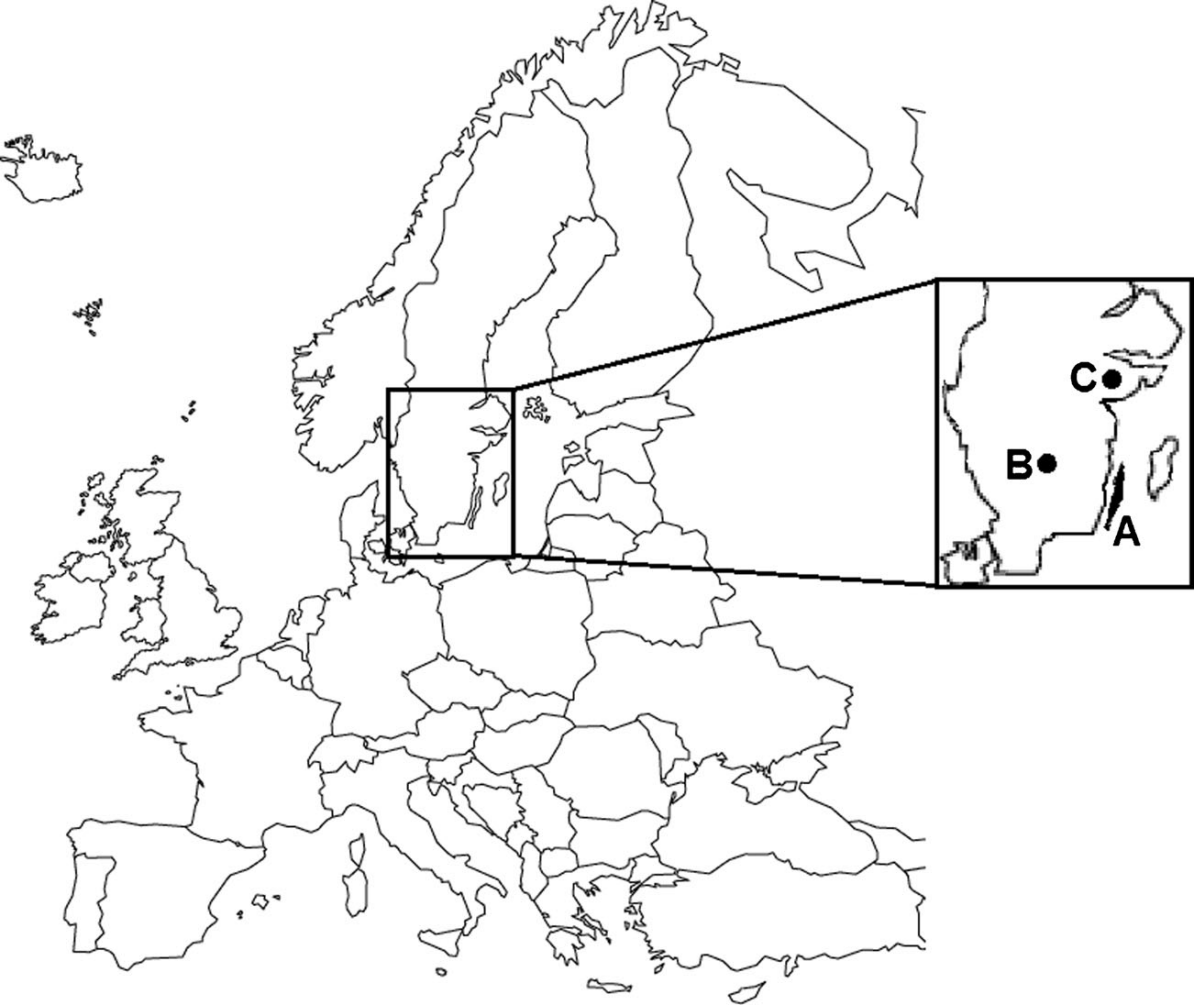
Map of Europe and Sweden with areas of moose sampling highlighted with *A* (Island of Öland), *B* (Province of Småland), and *C* (Province of Södermanland)

From 2007 to 2011, during the moose-hunting season (opening in the second week of October), reproductive organs and mandibles were collected from harvested female moose in three areas of the southern part of Sweden (Fig. 1). The sampling areas consisted of local moose management units located from 56°55.450′ N, 14°45.056′ E to 59°5.323′ N, 17°22.600′ E. Field laboratories for macroscopic examination of the reproductive organs were set up in the vicinity of the hunting areas, and hunters were asked to directly contact trained field personnel when a female moose was harvested. Collected samples were stored in coolers (at approximately +8 °C) during transport from the collection site to the field laboratory. Some samples (*n* = 35), that were collected without access to a field laboratory (from animals harvested later during the hunting season), were stored at −20 °C and transported frozen to the laboratory at the Department of Clinical Sciences in Uppsala, Sweden, where they were subsequently thawed and examined.

### Age determination

Moose age was determined by sectioning the first molar and counting of cementum layers (Wolfe 1969). In 22 of the 213 moose, age determination was not possible because hunters decapitated the animals before transport.

### Examination of reproductive organs

Ovarian structures were examined and the size and numbers of follicles (≥5 mm) and corpora lutea recorded. The presence of one or more corpora lutea was the criteria for ovulation. Uteri were weighed (ligaments and cervix not included) and cut open to macroscopically investigate mucous membranes and content, including embryos. Signs of early pregnancy (i.e., embryonic structures such as allantochorionic membranes, proper embryos, or remnants of embryos), as well as any abnormalities such as signs of uterus inflammation (discolored mucous membrane, presence of pus), were noted.

**Fig. 2:**
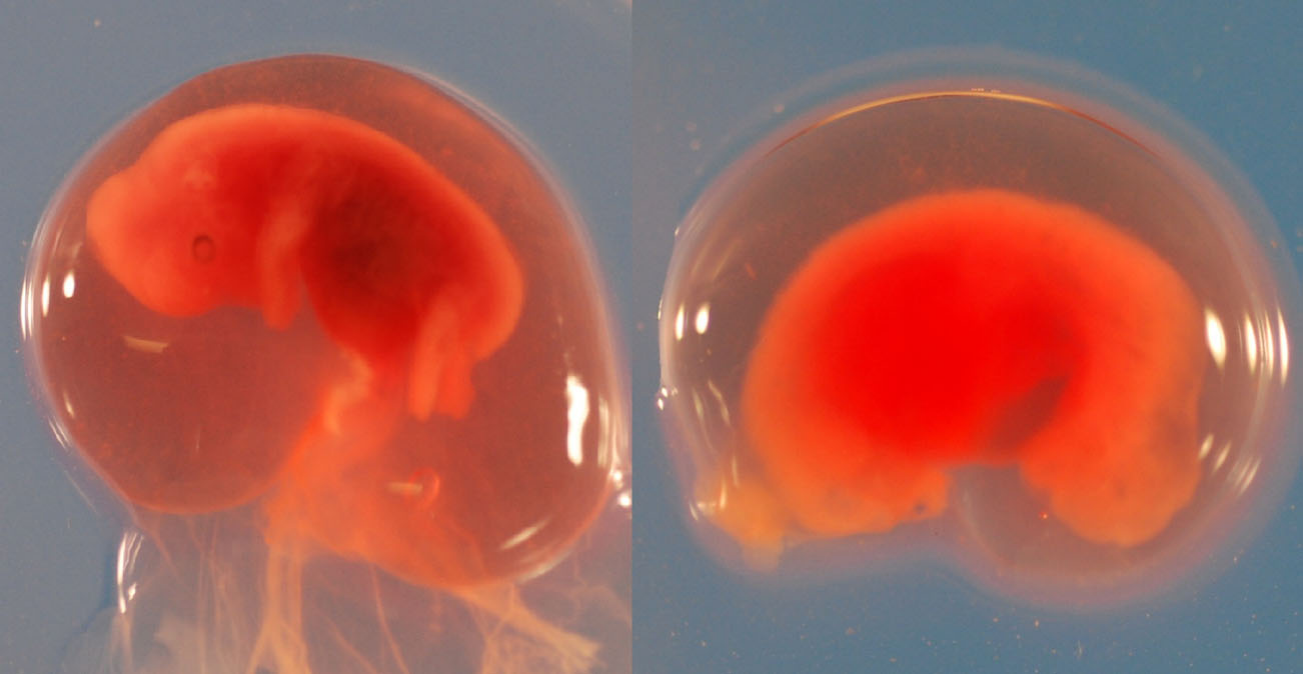
Viable (*left*) and nonviable (*right*) moose embryos
- Judged to be older than 2 weeks of pregnancy, i.e., longer and wider but not containing embryo proper or containing remnants of allantochorionic membranes (Fig. 3)

**Fig. 3:**
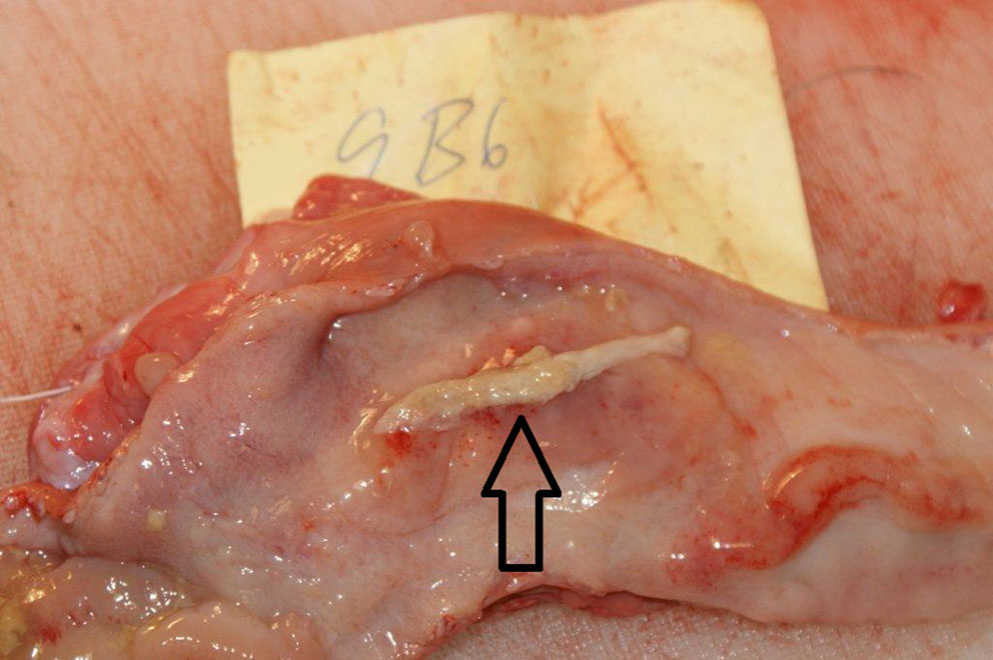
Remnant of allantochorionic membrane (*arrow*) in moose uterus

From uteri with signs of inflammation, samples for bacteriological culture were taken with a sterile swab and transported in Amies medium (Copan S.P.A., Brescia, Italy) to the National Veterinary Institute, Uppsala for routine aerobic culture on blood agar at 37 °C for 24 h. Tissue samples (0.5 × 1 cm) were taken from at least two sites of the uterus for microscopic examination to verify the macroscopic signs of inflammation (increased infiltration of inflammatory cells). The tissue samples were fixed in formalin solution (10 %), dehydrated, embedded in paraffin, and stained with hematoxylin–eosin. In order to determine reproductive status, ovarian and uterine findings were combined. Confirmation that conception had occurred included the presence of one or more corpora lutea, allantochorionic membranes, a proper embryo (Fig. 3), and/or remnants of embryos. The ratio between numbers of corpora lutea and embryos was calculated in order to attain the proportion of fertilized oocytes. To be classified as embryonic mortality, at least one of the following criteria had to be fulfilled:

- Malformed embryo with failure of organogenesis (uneven edges, no visible hind or forelimbs, misshaped head, absence of visible liver; Fig. 2)

### Data and statistical analyses

In all animals found to be pregnant, the total number of corpora lutea (CL_tot_) was calculated to assess the ovulation rate. To determine the rate of fertilized oocytes (conception rate), the total number of embryo-related structures (E_tot_) was divided by the total number of corpora lutea. Viable embryos without observed disorders were noted (E_norm_). The rate of embryonic mortality (EM) was determined by dividing all embryonic structures judged to be nonviable (E_malf_) with E_tot_. To determine the rate of unfertilized oocytes, the total number of embryo-related structures (E_tot_) was subtracted from the total number of corpora lutea (CL_tot_). Pregnancy outcome (mean number of embryos per pregnant female) was determined by subtracting the rate of embryonic mortality and the rate of unfertilized oocytes from the ovulation rate.

An exploratory analysis of multiple risk factors affecting the presence of embryonic mortality was performed using a logistic regression in R (R Core Team 2013, Table 4). Crude and adjusted odds ratios (OR) with 95 % confidence intervals were calculated to identify which factors (age of female, sampling area, sampling year, presence of twin embryos, presence of endometritis, and number of corpora lutea) were associated with embryonic mortality. Crude OR is an estimation of one specific factor without consideration and possible confounding effects of other factors. The adjusted OR is estimated with consideration of other factors (stated previously) included in the model. In addition, statistical significance calculations (*P* values) between the different risk factors were performed (Table 4).

## Results

### Sampled moose

In total, 213 female moose were sampled. The majority of the samples (91.1 %, *n* = 194) were collected from Oct 10 to Nov 11, and the remaining samples (*n* = 19) were collected from Nov 12 to Dec 12. The mean age of sampled female moose was 4.2 years (range, 1.5–18.5).

### Reproductive organ examinations

The majority of the investigated moose (67.3 %, *n* = 142) had ovulated (corpora lutea present, Table 1), and these animals were then included in the further analyses. A total of 53 (37.3 %) females that had ovulated fulfilled the criteria for being pregnant. The mean ovulation rate for the pregnant females was 1.49, and the mean rate of fertilized oocytes was 1.43 per capita. The proportion of unfertilized oocytes (ova loss) in pregnant moose was 6.3 %, the twinning rate was 43.4 %, and one triple ovulation (0.7 %) was noted. Embryonic mortality was observed in 16 individuals (30.9 % of pregnant moose), carrying a total of 19 (25 % of all embryos) malformed embryos, or remnants thereof (Table 2). The total loss of potential offspring due to unfertilized oocytes and embryonic mortality was 27.5 %, i.e., the discrepancy between the ovulation rate of pregnant females (1.49) and the early pregnancy rate per female (1.08).

**Table 1:** Sampling area, time of sampling, and age and ovulation rates in 142 samples of female moose (*Alces alces*) that had ovulated, from 2007 to 2011

Area	Time range of sample collection	Number of samples	Mean age (range)	Mean ovulation rate (range)
A (Öland)	Oct 10–Nov 11	64	5.1 (1.5–18.5)	1.39 (1–3)
	Nov 12–Dec 12	1	4.5	2 (2)
B (Småland)	Oct 10–Nov 11	43	3.9 (1.5–14.5)	1.1 (1–2)
	Nov 12–Dec 12	0	–	–
C (Södermanland)	Oct 10–Nov 11	33	3.3 (1.5–7.5)	1.58 (1–2)
	Nov 12–Dec 12	1	5.5	2 (2)
All	Oct 10–Dec 12	142	4.4 (1.5–18.5)	1.35 (1–3)

**Table 2:** Sampling area, age, ovulation rate, proportion of fertilized oocytes, twin pregnancy, and number and proportion of malformed and normal embryos in 53 pregnant female moose (*Alces alces*), 2007–2011

Area	*N*	Mean age	CL_tot_^a^	Mean ovulation rate	Number. of embryonic structures^b^	Rate of fertilized oocytes (%)	Number and rate (%) of twin pregnancy	E_malf_^c^	E_norm_^d^	Number of unfertilized oocytes (%)	Number of normally developed embryos per female
A	17	4.2	24	1.41	24	1.41 (100)	7 (41.1)	10	14	0 (0)	0.82
B	8	7.7	12	1.50	12	1.50 (100)	4 (50.0)	1	11	0 (0)	1.38
C	28	3.5	45	1.61	40	1.43 (89)	12 (42.9)	8	32	5 (11.1)	1.14
All	53	4.3	81	1.49	76	1.43 (96)	23 (43.4)	19	57	5 (6.3)	1.08

^a^Sum of corpora lutea in all examined animals

^b^Normally developed and malformed embryonic structures

^c^Total number of malformed embryos

^d^Total number of normally developed embryos

Among the pregnant females, 12 (23.1 %) had macroscopical signs of inflammation of the inner mucous membrane of the uterus (endometritis), which was confirmed by microscopical examination, and eight of those animals also had embryonic mortality (Table 3). Hence, 50 % of the females with embryonic mortality also had endometritis (Table 3). Aerobic cultivation of bacteriological samples (*n* = 8) from uteri was negative. The risk of embryonic mortality increased with presence of endometritis (*P* < 0.001). Also, the risk of endometritis increased with female age (*P* = 0.003; Table 4). In addition, a temporal effect on the presence of embryonic mortality (*P* = 0.009) was observed.

**Table 3:** Information on 16 pregnant female moose with embryonic mortality

Area	Year	Date	Age	Number of corpora lutea	Number of malformed embryos	Number of normally developed embryos	Number of non fertilized oocytes	Confirmed endometritis (1 = yes, 0 = no)
C	2010	–^a^	14.5	1	1	0	0	0
B	2009	Oct 13	1.5	1	1	0	0	1
B	2009	Oct 13	4.5	2	2	0	0	1
B	2010	Oct 13	2.5	1	1	0	0	1
B	2010	Oct 13	8.5	2	2	0	0	1
B	2010	Oct 11	–^a^	2	2	0	0	1
B	2011	Oct 11	17.5	2	1	1	0	1
B	2011	–^a^	–^a^	2	1	1	0	0
A	2007	Nov 11	1.5	1	1	0	0	0
A	2007	Nov 3	1.5	1	1	0	0	0
A	2007	Nov 1	2.5	2	1	0	1	0
A	2007	Nov 3	2.5	2	1	1	0	0
A	2007	Nov 3	4.5	1	1	0	0	0
A	2009	Nov 7	4.5	2	1	0	1	1
A	2010	Oct 30	6.5	2	1	1	0	1
A	2011	Oct 29	2.5	1	1	0	0	1
A–C	2007–2011		5.4	25 (1.56)	19 (1.19)	4 (0.25)	2 (0.13)	8 (0.5)

*A* Island of Öland, *B* Province of Småland, *C* Province of Södermanland

^a^Information not available

**Table 4:** Results of a logistic regression model with factors associated with presence of embryonic mortality in pregnant female moose from Sweden sampled from 2007 to 2011

Variable	Crude^a^ OR (95 % CI)	Adjusted^b^ OR (95 % CI)	*P* value (Wald's test^c^)	*P* value (LR test^d^)
Age	1.19 (0.95–1.50)	1.64 (1.11–2.41)	0.012	0.003
Area	0.56 (0.26–1.21)	0.43 (0.11–1.78)	0.246	0.224
Sampling year	1.06 (0.65–1.73)	0.27 (0.08–0.89)	0.031	0.009
Twins	0.83 (0.22–3.20)	0.17 (0.01–3.52)	0.253	0.253
Endometritis^e^	9.72 (1.92–49.11)	157.57 (5.05–4,919.25)	0.004	<0.001
No. of corpora lutea	1.01 (0.27–3.78)	2.76 (0.17–43.50)	0.471	0.477

^a^The odds ratio of one specific factor without consideration of confounding effects of other factors

^b^The odds ratio of one factor, with consideration of confounding effects of other factors

^c^The *P* value based on the sample estimate

^d^Likelihood ratio test

^e^Inflammation of the inner mucous membrane of the uterus

## Discussion

### Ova loss and embryonic mortality

Here, we show that reproductive failure in early pregnancy, commonly described as ova loss, is common in Scandinavian moose. We also show that the major part of the loss was attributed to the embryonic mortality and not to the actual loss of an oocyte. In accordance with domestic animal reproductive terminology, we also suggest that ova loss is to be defined as when an oocyte fails to become fertilized by a sperm (conception failure), or when a fertilized oocyte due to impaired quality is terminated in very early pregnancy (approx. <12 days since conception) (Schillo 2009). Embryonic mortality (or embryo loss) is defined as mortality after this period, and can occur until the embryo becomes a fetus, which occurs when the fetus has identifiable features of a given species (Schillo 2009). In moose, embryonic mortality has been discussed as a plausible cause of discrepancies between ovulation rate and embryo/fetal rate (Edwards and Ritcey 1958; Markgren 1969; Pimlott 1959; Schwartz and Hundertmark 1993; Simkin 1965). Markgren (1969) noted a 19 % ova loss in Scandinavian moose, and Schwartz and Hundertmark (1993) estimated a 9.3 % loss in Alaskan moose (*Alces alces gigas*). Few reports on the detection of one or more nonviable embryos exist to date, most likely due to practical difficulties in repeated testing (by rectal ultrasonography) of female moose in the wild. Another cause, which may contribute to an underreporting of the event, could be difficulties in differentiating a nonviable from a viable embryo when the embryonic membranes are intact. Hence, previous reports on ova loss may include undetected events of embryonic mortality. Also, thorough examination of an embryo in order to detect failure of organogenesis may not have been performed, and the embryo has been labeled as normal/viable instead of the opposite. Testa and Adams (1998) did however detect a presumed dead embryo using rectal ultrasonography in immobilized Alaskan moose, and one case of fetal mortality was reported by Schwartz and Hundertmark (1993). In elk (*Cervus canadensis*), Trainer (1971) described embryonic mortality on two occasions (2.5 % of 79 examined females), and Woolf and Harder (1979) also reported a low (1.6 % of 60 examined animals) presence of embryonic mortality in white-tailed deer (*Odocoileus virginianus*). Furthermore, embryonic mortality has been diagnosed in captive fallow deer (*Dama dama*), where Willard et al. (1998) used repeated hormone analyses (progesterone, estrone sulfate, pregnancy-specific protein) and transrectal ultrasonography to detect embryonic mortality in 2 out of 10 pregnant females. In free-ranging caribou (*Rangifer tarandus*), Russell et al. (1998) reported embryonic mortality in 8.5 % (12/141) of the investigated radio-collared females, using repeated hormone analyses (progesterone, pregnancy-specific protein). Using transrectal ultrasonography after mating, and studying subsequent parturition rates, Gómez-Nieto et al. (2011) stated that 13.0 % (6/46) of captive red deer (*Cervus elaphus*) most likely had experienced embryonic mortality. Embryonic mortality and lack of fertilization of oocytes are well-known occurrences in domestic species, e.g., cattle (Diskin et al. 2011; Hanly 1961; King 1991), sheep (Cognie et al. 1975; Dutt and Simpson 1957; Vázquez et al. 2009; Viñoles et al. 2012), goats (Armstrong and Evans 1983; Gonz et al. 2004; Shelton 1978), pigs (Dziuk 1968; Geisert et al. 2007; Soede et al. 1994), and horses (Ball et al. 1986; Dippert et al. 1994; Newcombe and Cuervo-Arango 2011), and underlying causes of may be infectious (bacteria, virus, parasites, fungi) or of genetic, endocrine, and environmental origin (Diskin et al. 2011).

### Inflammation of the uterus

Endometritis, defined as inflammation of the inner mucous membrane in the uterus, is a condition observed in domestic animals. In dairy cattle, it often occurs postpartum, where bacteria of different species enter the uterus and cause an infection. The association between endometritis and embryonic mortality was considerable, as 50 % (8/16, Table 2) of all cases of embryonic mortality also was accompanied by microscopically confirmed endometritis. In addition, the risk of endometritis (and embryonic mortality) seems to increase with age. It is known from domestic animals that embryos do not survive in uteri with endometritis. In the present study, the aerobic culturing of samples from uteri was negative. Hence, the cause of endometritis in the sampled female moose was not fully elucidated but may include an anaerobic bacterial infection introduced at mating or after mating. In the latter case, bacteria can ascend from the vagina into the uterus in females where the neck of the uterus (cervix uteri) does not function as a proper barrier. To our knowledge, endometritis has never been reported in wild cervids. McShea et al. (1997) reported endometritis in a captive white-tailed deer, which had been given immunocontraceptives. The considerably high presence of endometritis in moose in the present study indicates that this condition most likely is underdiagnosed in other ungulates.

### Moose productivity estimation

The use of estimation of productivity (reproductive rates) of moose populations by ovulation rates is common in Scandinavia and North America (Ferguson et al. 2000; Saether and Haagenrud 1985; Sand and Cederlund 1996; Simkin 1965). However, based on the high rate of early reproductive failure seen in the present study, an overestimation of future numbers of offspring is possible. Though rarely reported, (Schwartz and Hundertmark 1993) fetal loss must also be considered, which if occurring, affects future offspring numbers in a population.

### Causes of embryonic mortality

The reasons for the embryonic mortality in the present study are most likely multifactorial and could include impaired quality of the oocyte or spermatozoa at fertilization as well as an inadequate maternal recognition of pregnancy. In sheep, the rate of very early losses (until the early embryonic stage) is approximately 5–10 %, which is considered acceptable from a management point of view (Diskin and Morris 2008). However, in lactating dairy cows, the loss rate has been reported to be around 20 %, where the marked increase in milk yield over the past 40 years, has been accompanied by a decrease in fertility (Diskin and Morris 2008). Understandably, this relationship is not applicable for moose in the wild, and hence the causes are of another nature. Uterine malformations as a cause of embryo mortality were not identified in the present study, and chromosomal aberrations, which can lead to early embryonic mortality (Diskin and Morris 2008), were not investigated in the dead embryos of the present study. A temporal effect (year of sampling) of embryonic mortality was shown in the present study, where the proportion of females with embryonic mortality differed between years. This effect is likely due to annual differences in the age structure of sampled moose.

## Conclusion

In conclusion, we verified our hypothesis stating that loss of ova is not only attributed to unfertilization of oocytes (ova loss) but also dependent on embryo mortality and survival. Also, we verified the second hypothesis and showed that there is a discrepancy between ovulation rates and embryo survival rates.

Ovulation rates in moose are substantially higher than the actual proportion of normally developed embryos in the uterus due to embryonic mortality and unfertilized oocytes. This should be taken into consideration when trying to estimate moose population productivity for management purposes. Reproductive tract investigations solely focusing on the presence of corpora lutea may lead to misinterpretations of moose productivity.
